# The antagonistic potential of peanut endophytic bacteria against *Sclerotium rolfsii* causing stem rot

**DOI:** 10.1007/s42770-022-00896-x

**Published:** 2022-12-27

**Authors:** Liangliang Li, Jiwen Wang, Dehai Liu, Lei Li, Jing Zhen, Gao Lei, Baitao Wang, Wenling Yang

**Affiliations:** Institute of Biology Co., Ltd., Henan Academy of Sciences, Key Laboratory of Microbial Engineering of Henan, Zhengzhou, 450008 China

**Keywords:** *Bacillus* sp.,·*Burkholderia* sp., *Sclerotium rolfsii*, Peanut endophytic bacteria, Defense enzyme

## Abstract

Peanut stem rot caused by *Sclerotium rolfsii* Sacc. is the most common disease of peanut worldwide and has become increasingly serious in recent years. This study is aimed at obtaining peanut endophytic bacteria with high antagonistic/protective effects against peanut stem rot. In total, 45 bacterial strains were isolated from healthy peanut plants from a severely impacted area. Of these, 6 exhibited antagonistic activity against *S. rolfsii*, including F-1 and R-11 with the most robust activity with an inhibition zone width of 20.25 and 15.49 mm, respectively. These two were identified as *Bacillus* sp. and *Burkholderia* sp., respectively, based on morphological, physiological, and biochemical characteristics and 16S rDNA sequencing. To the best of our knowledge, this is the first study to report the *Burkholderia* sp. antagonistic effect on *S. rolfsii* as a biological control agent for peanut stem rot. Their culture filtrates potently inhibited the hyphal growth, sclerotial formation, and germination of *S. rolfsii*. Also, the strain-produced volatile compounds inhibited the fungal growth. Pot experiments showed that F-1 and R-11 significantly reduced the peanut stem rot disease with the efficacy of 77.13 and 64.78%, respectively, which was significantly higher compared with carbendazim medicament (35.22%; *P* < 0.05). Meanwhile, F-1 and R-11 improved the activity of plant defense enzymes such as phenylalaninase (PAL), polyphenol oxidase (PPO), and peroxidase (POD) enhancing the systemic resistance of the peanut plants. This study demonstrated that *Bacillus* sp. F-1 and *Burkholderia* sp. R-11, with a strong antagonistic effect on *S. rolfsii*, can be potential biocontrol agents for peanut stem rot.

**Supplementary Information:**

The online version contains supplementary material available at 10.1007/s42770-022-00896-x.

## Introduction

Peanut (*Arachis hypogaea* L.) is one of the most important oil crops in the world and plays an important role in the world oil production [1, 2]. However, it is highly prone to diseases during planting, especially by stem rot induced by *Sclerotium rolfsii* Sacc (SR). This has been an important factor restricting the output and quality of peanuts worldwide [3]. *Sclerotium rolfsii,* with widespread hosts, can infect >500 plants such as peanut, pepper, oil-tea camellia, and *Atractylodes macrocephala*. SR is a severe soil-borne pathogenic fungus, especially in warm and humid areas [4]. The pathogen hibernates in the soil or on the diseased body as a sclerotium. In the subsequent year, the sclerotium germinates to produce mycelium, the primary infection source, which gets transmitted through soil, flowing water, insects, and germ-bearing seeds [5, 6]. Sclerotium can survive for several months to years in soil and therefore is difficult to control. Currently, peanut stem rot prevention/treatment mainly depends on crop rotation, breeding resistant varieties, and chemicals. However, none of them is fully effective [7]. Meanwhile, the chemicals pollute the environment, destroy the ecological balance, and induce pathogen resistance [8, 9]. Therefore, the biological prevention/treatment of peanut stem rot is gaining much attention.

Currently, *Bacillus* spp., *Pseudomonas* spp., *Streptomyces* spp., *Trichoderma* spp., and arbuscular mycorrhizal fungi are the main biocontrol species against peanut stem rot [10–18]. Lu et al. [19] reported that *B. amyloliquefaciens* 41B-1 could inhibit the growth rate of SR mycelium, destroy the mycelium structure, reduce the number of sclerotium, and survive inside the root system for a long time inducing plant systemic resistance. Kishore et al. [20] screened 12 *P. aeruginosa* strains from 393 bacteria that showed an inhibition rate of 32.0-74.0% against SR mycelium. The GSEl8 and GSEl9 culture filtrates effectively inhibited SR-produced enzymes polygalacturonase and cellulase. Greenhouse experiments showed that inoculation with GSEl8 and GSEl9 reduced 54 and 58% of SR-induced dead seedlings, respectively. Karthikeyan et al. [21] compared biocontrol performances of 3 *T. viride* strains and 1 *T. harzianum* strain against SR. The *T. viride* strain inhibited the growth of SR mycelium (by 69.4%) and sclerotium. They found that 5 g of *T. viride* strain per kilogram of soil reduced peanut stem rot morbidity to 3.75%, which was significantly lower than the control group (40.0%). Figueredo et al. [22] found that *Bacillus* sp. CHEP5 could improve the phenylalanine ammonia-lyase activity of peanut plants producing systemic resistance against SR*.* Under artificial culture, many strains of the above bacteria inhibited the growth of SR mycelium, germination of sclerotium, and stem rot. However, there are rare reports of successful large-scale production and applications [4]. Thus, it is an urgent need to find novel biocontrol strains.

Plant endophytic bacteria remain distributed in different plant tissues for nutrients and protection from harsh external environments including sunlight, ultraviolet rays, wind, and rain. Having a stable ecological environment, plant endophytic bacteria can offer unique advantages in disease prevention and growth promotion [23, 24]. In this study, endophytic bacteria were isolated from different tissues (root, stem, leaf, and flower) of peanut plants identifying the two most effective strains (F-1 and R-11) against SR. Their control effects on peanut stem rot were investigated in detail.

## Materials and methods

### Pathogen

SR was isolated from a diseased peanut plant in Runan County, Henan Province [25] and stored in Key Laboratory of microbial engineering of Henan, Zhengzhou, China.

### Isolation of peanut endophytic bacteria

Peanut samples were collected from Runan County, Henan Province, China; the geographical coordinates were 114° E, 32° N. The healthy peanut plants (cv. Yuhua 37) were collected in the peak flowering period, with an intact root system, and then immediately put into an icebox for transport back to the laboratory. The surface soil on the peanut plant was removed with plenty of sterile water, and then, the tissue surface was disinfected. Briefly, the different tissue samples of peanut were rinsed with sterile water for 30 s, then rinsed with 75% alcohol for 2 min, followed by soaking in 2.5% sodium hypochlorite solution for 5 min, and then finally rinsing with a large amount of sterile water thrice. Finally, the tissue-specific isolation of endophytic bacteria was performed after primary flushing solution coating of nutrient agar (NA) plate ensuring sterility. Under a sterile environment, root, stem, leaf, and flower tissues of peanut were separated by scissors, added to sterile normal saline, and then ground into respective suspensions. After appropriate dilution, suspensions were coated on the NA plate and cultured at 30 °C for 48 h. The bacterial colonies with significant morphological differences on the NA plate were selected and purified 3 times by the streak plate method. Finally, purified single colonies were transferred to respective NA slants for storage at 4 °C.

### Screening of antagonistic bacteria

Antagonistic bacteria of SR were identified by the dual culture test. Specifically, the SR mycelial disk, with a diameter of 5 mm, was point-inoculated in the middle of a potato dextrose agar (PDA) plate (diameter 90 mm). Next, the NA culture medium activated peanut endophytic bacteria was streak-inoculated on both sides 30 mm away from the plate center. The PDA plate inoculated with SR alone was set up as the control group; each group had three replicates. All plates were incubated at 25 °C. The width of inhibition zone from the edge of fungal colony to the edge of bacterial colony was measured, while the control group showed growth in full plate.

### Identification of antagonistic bacteria

The screened antagonistic bacteria (F-1 and R-11) were identified based on morphological, physiological, and biochemical characteristics as reported elsewhere [26]. Furthermore, these were identified by 16S rDNA sequencing. The genomic DNA of antagonistic bacteria was extracted with a Bacterial Genomic DNA Extraction Kit (Solarbio, Beijing, China) and amplified with bacterial 16S rDNA primers: 27f: 5′-AGAGTTTGATCCTGGCTCA-3′, 1492r: 5′-GGTTACCTTGTTACGACTT-3′ [27]. The PCR reaction system (50 μL) included 1 μL 27F (10 μmol L^-1^), 1 μL 1492R (10 μmol L^-1^), 2 μL DNA template (50 μg mL^-1^), 25 μL 2 × PCR Mix, and 21 μL ddH_2_O. PCR conditions were as follows: 94 °C for 3 min, 33 cycles at 94 °C for 30 s, 55 °C for 30 s, and 72 °C for 1.5 min, followed by a final extension at 72 °C for 10 min. The amplified products were analyzed by 1% agarose gel electrophoresis and sequenced by the Beijing Genomics Institute, China. The sequences obtained were compared for homology with the reference sequences in GenBank using BLASTN, and the neighbor-joining phylogenetic trees were constructed using MEGA 6.05 based on the 16S rDNA sequence.

### Antagonistic effect of culture filtrate of antagonist bacterial isolates on SR

Activated F-1 and R-11 were inoculated into Erlenmeyer flasks containing 100 mL nutrient broth (NB) at 200 rpm and 30 °C. After 3 d, the bacterial cultures were centrifuged at 8000 g and 4 °C for 20 min. The supernatant was filtered through a 0.22 μm microporous membrane to obtain the sterile culture filtrate. The filtrates were diluted with sterile water 2 and 4 times and then mixed with PDA in the proportion 1 : 4 (v/v). Next, SR mycelial disk, with a diameter of 5 mm, was point-inoculated in the middle of a PDA plate. A negative control group was set up with the NB culture medium instead of culture filtrate. Each group had three replicates and was incubated in an isothermal incubator at 25 °C. The control group covered the whole plate and the colony diameter was measured. Then, the inhibition rates of different dilutions of bacterial culture filtrate (stock solution, 2-fold, and 4-fold dilutions) were investigated on the growth of SR mycelium.

$$\textrm{Inhibition}\ \textrm{rate}\ \left(\%\right)=\left[\ \left(D-d\right)/\left(D-5\right)\right]\times 100.$$

where *D* is the diameter of SR in the control PDA, *d* is the diameter of SR in the treatment PDA, and the 5 is the diameter of the inoculated plug of SR.

After a continuous culture for 30 d, the number of mature sclerotium formed on each plate was recorded. The effects of F-1 and R-11 culture filtrates on the sclerotium of SR were evaluated.

Sclerotia were kept in 75% ethanol for 2 min, 2.5% sodium hypochlorite solution for 3 min, and then 75% ethanol for 30 s. Finally, these were rinsed with sterile water thrice for surface disinfection. The surface-sterilized sclerotia were placed on the PDA plate containing antagonistic bacteria culture filtrate and the plate was cultured at 25 °C. The PDA plate added with NB culture medium was used as the control group. Twenty sclerotia were placed on each plate and the germination was recorded after 72 h to calculate the sclerotium germination inhibition rates. Each group had three replicates with 5 plates each, a total of 100 sclerotia.

$$\textrm{Inhibition}\ \textrm{rate}\ \left(\%\right)=\left(\textrm{number}\ \textrm{of}\ \textrm{non}-\textrm{germinating}\ \textrm{sclerotium}/\textrm{total}\ \textrm{number}\ \textrm{of}\ \textrm{sclerotium}\right)\times 100$$

### Inhibitory effect of volatile compounds released by antagonistic bacteria on SR

Activated F-1 and R-11 were inoculated into the Erlenmeyer flasks containing 100 mL NB culture medium at 30 °C and 200 rpm for 24 h. The cultures were adjusted to 1.0 × 10^9^ cfu mL^-1^ with sterile water and evenly mixed with 50 °C NA culture medium (1:20). Next, the culture plate was inverted. Fresh SR, with a diameter of 5 mm, was inoculated into the center of a PDA plate. Then, the PDA plate was flipped onto the treated NA plate, and the plates’ edges were sealed with double-layer sealing film. The plates were kept in an isothermal incubator at 25 °C. The NA plate added with the same amount of sterile water was regarded as a negative control group. Each group had three replicates. Mycelium fully covered the control group PDA plate. The diameter of the pathogen colony of different treatment groups was measured by the cross method to measure the inhibition rate as described above.

### Pot experiment

The peanut seeds of uniform size and plumpness were soaked in sterile water and then incubated at 30 °C for 24 h. The sprouted peanut seeds were seeded into 200 g of sterilized natural soil in a 12 cm × 15 cm plastic basin. The pot experiment was performed under greenhouse conditions at 60% humidity, 25 °C, and 16/8 h of light and darkness. After sowing for 30 d, peanut seedlings with consistent growth were selected for the pot experiment; one peanut plant was retained in each pot. Group 1 (negative control group) was without pathogen and biocontrol bacteria. Group 2 (positive control group) had SR and was inoculated with 50 mL of sterile water. Group 3 (fungicide control group) was inoculated with the pathogen and 50 mL of 50% carbendazim wettable powder, 1000 times diluted inoculation. Group 4 (F-1 group) was inoculated with the pathogen and 50 mL of 3 d grown F-1 fermentation broth. Group 5 (R-11 group) was inoculated with the pathogen and 50 mL of 3 d grown R-11 fermentation broth. Each treatment group comprised 10 peanut plants and three replicates. After 28 d, the morbidity and disease index of peanut plants was examined to evaluate the prevention and treatment effects of different treatments on peanut stem rot. The disease severity was graded as follows. Level 0: peanut was healthy and free of disease spots; level 1: yellowed peanut leaves and the infected parts with wilting and other symptoms accounted for <25% of the whole plant; level 2: yellowed peanut leaves and the infected parts with wilting and other symptoms accounted for 25-50% of the whole plant; level 3: yellowed peanut leaves and the infected parts with wilting and other symptoms accounted for 50-75% of the whole plant; and level 4: yellowed peanut leaves and the infected parts with wilting and other symptoms accounted for >75% of the whole plant, and the plant withered.

$$\textrm{Morbidity}\ \left(\%\right)=\left(\textrm{number}\ \textrm{of}\ \textrm{diseased}\ \textrm{plants}/\textrm{total}\ \textrm{number}\ \textrm{of}\ \textrm{plants}\right)\times 100$$

$$\textrm{Disease}\ \textrm{index}=\sum \left(\textrm{number}\ \textrm{of}\ \textrm{diseased}\ \textrm{plants}\ \textrm{at}\ \textrm{all}\ \textrm{levels}\times \textrm{representative}\ \textrm{value}\textrm{s}\ \textrm{at}\ \textrm{those}\ \textrm{levels}\right)\times 100/\left(\textrm{total}\ \textrm{number}\ \textrm{of}\ \textrm{investigated}\ \textrm{plants}\times \textrm{highest}\ \textrm{representative}\ \textrm{value}\right)$$

$$\textrm{Control}\ \textrm{efficacy}\ \left(\%\right)=\left[\left(\textrm{positive}\ \textrm{control}\ \textrm{group}\ \textrm{disease}\ \textrm{index}-\textrm{disease}\ \textrm{index}\ \textrm{of}\ \textrm{the}\ \textrm{treatment}\ \textrm{group}\right)/\textrm{positive}\ \textrm{control}\ \textrm{group}\ \textrm{disease}\ \textrm{index}\right]\times 100$$

### Effects of antagonistic bacteria on peanut defense enzymes

After pathogen inoculation for 3, 7, 14, 21, and 28 d, 0.4 g of functional leaves expanded at the top of the main peanut stem was ground in the liquid nitrogen and then added 4 mL 0.1 M borate buffer with 5 mM β-mercaptoethanol. The mixture was centrifuged at 4 °C and 8000 g for 10 min. The supernatant was enzyme extract used to measure the related indices of induced plant systemic resistance, such as phenylalaninase (PAL), polyphenol oxidase (PPO), and peroxidase (POD).

Phenylalaninase (PAL) activity was assayed following the method described by Chen et al. [28]. The activity was measured with 100 μL enzyme extract, 300 μL borate buffer, 500 μL L-phenylalanine (0.1 M), and 1 mL distilled water. This mixture was incubated at 30 °C for 30 min and the reaction was terminated by the addition of 500 μL 1 M trichloroacetic acid. The activity was measured at 290 nm and expressed by the change of absorbance per min per g fresh weight.

Polyphenol oxidase (PPO) activity in plant tissue was measured according to Aquino-Bolaños et al. [29]. The activity was measured with 1 mL of 0.1 M citrate–phosphate buffer, 500 μL of catechol solution (2 mM) as a substrate, and 100 μL of enzyme extract. The mixture was kept at 25 °C and absorbance was read at 410 nm in 60-sec intervals for 5 min. The activity was expressed by the change in absorbance per min per g fresh weight.

Peroxidase (POD) activity was estimated using the method described by Han *et al.* [30]. The reaction was carried out at 25 °C with 50 μL enzyme extract, 2 mL phosphate buffer (50 mM), 200 μL guaiacol (20 mM), and 100 μL of hydrogen peroxide. The absorbance was recorded at 470 nm. The peroxidase activity was expressed by the change in absorbance per min per g fresh weight.

### Statistical analysis

Each experiment was carried out at least three times. All data were expressed as mean ± SD and analyzed by one-way analysis of variance at the 5% level. Statistical differences between treatments were analyzed by Duncan’s multiple-range test at 5% significance level.

## Results

### Isolation and screening of antagonistic bacteria

In total, 45 bacteria strains were isolated from peanut plants, including 23 from root (R-1 ~ R-23), 10 from the stem (S-1 ~ S-10), 8 from leaf (L-1 ~ L-8), and 4 from flower (F-1 ~ F-4). Interestingly, 6 bacterial strains showed vigorous antifungal activity against SR with inhibition zone widths of >10 mm (Table 1). Among them, F-1 and R-11 were found to be the best with the inhibition zone widths of 20.25 and 15.49 mm, respectively (Fig. S1).

**Table 1:** Antagonistic activity of peanut endophytic bacteria against *Sclerotium rolfsii*

Strain	Inhibition zone width (mm)
R-11	15.49 ± 0.53^b^
R-23	11.67 ± 0.61^c^
S-2	14.21 ± 0.43^bc^
S-8	10.86 ± 0.29^c^
F-1	20.25 ± 0.51^a^
L-4	12.91 ± 1.03^c^

The inhibition zone width was measured from the edge of fungal colony to the edge of bacterial colony. Different lowercase letters in the same column indicate a significant difference between the treatments (*P* < 0.05)

### Identification of antagonistic bacteria

Strain F-1 was cultured on the NA plate for 48 h. The colony surface was dry, wrinkled, opaque, and yellowish, with irregular or nearly circular edges. The thalli were characterized as rod, single-cell, and spore. Likewise, strain R-11 was cultured and the colonies were pale yellow, uplifted with neat edges. The thalli were characterized as rod shape and flagella. Physiological and biochemical characteristics of F-1 and R-11 are listed in Table S1.

**Fig. 1:**
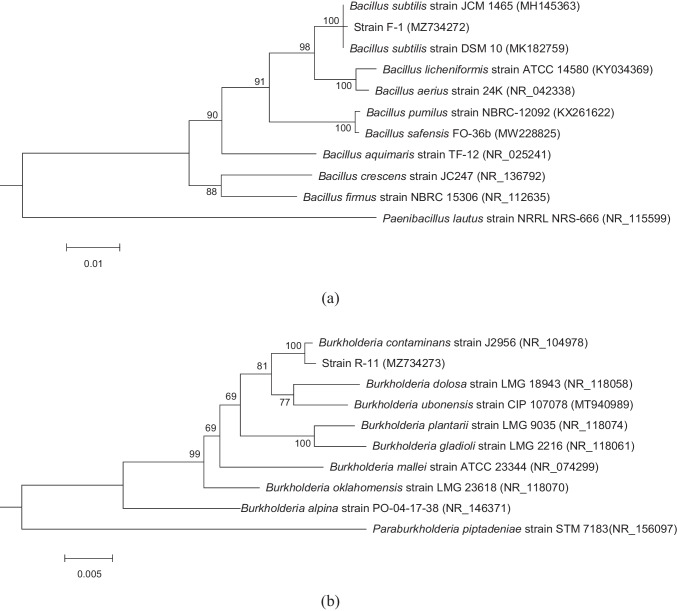
Neighbor-joining 16S rRNA gene phylogenetic tree of (**a**) F-1 and (**b**) R-11. The corresponding GenBank accession numbers are shown in the brackets. The bootstrap values are shown at the respective nodes

The 16S rDNA sequence lengths of F-1 and R-11 were 1333 and 1330 bp, respectively. The sequences had been deposited in GenBank with the accession number MZ734272 and MZ734273, respectively. Blast comparison results showed that F-1 was 99.92% homologous to *B. subtilis* strain JCM 1465 (accession No. MH145363) and *B. subtilis* strain DSM 10 (accession no. MK182759). A phylogenetic tree, constructed based on 16S rDNA sequences, showed a close genetic relationship between F-1 and *B. subtilis* (Fig. 1a). R-11 was 99.85% homologous to *Burkholderia contaminans* J2956 (accession No. NR_104978). The constructed phylogenetic tree showed a close genetic relationship between the two (Fig. 1b). According to morphological, physiological, and biochemical characteristics and 16S rDNA sequence analysis, F-1 and R-11 were identified as *Bacillus* sp. and *Burkholderia* sp., respectively. Both strains were collected as CGMCC No. 20856 and 21139 by the China General Microbiological Culture Collection Center.

### Effects of antagonistic bacteria culture filtrate on the growth of SR

F-1 and R-11 culture filtrate showed a significant antagonistic effect on the growth of SR mycelium with inhibition rates (stock solutions) of 91.66% and 90.56%, respectively (Table 2). With the increase of dilution rate, the antifungal activity of the filtrates decreased gradually; however, it remained >35% for quadruple dilutions.

**Table 2:** The effects of culture filtrate of antagonistic bacteria F-1 and R-11 over *Sclerotium rolfsii* growth

Strain	Dilution times	Inhibition rate of radial growth (%)	Number of mature sclerotia/piece	Ungerminated sclerotia (%)
F-1	Original (stock)	91.66 ± 3.38	22.33 ± 1.70	96.33 ± 2.05
	2 times	61.84 ± 4.21	45.00 ± 3.27	65.67 ± 2.87
	4 times	35.47 ± 2.67	63.67 ± 2.05	43.00 ± 3.74
R-11	Original	90.56 ± 4.28	12.00 ± 1.63	88.67 ± 2.05
	2 times	73.86 ± 3.67	28.00 ± 2.16	47.33 ± 2.49
	4 times	42.34 ± 3.59	40.67 ± 2.05	27.67 ± 1.70
CK (control)	Original	0	91.67 ± 4.03	2.67 ± 0.94

Compared with the control group, F-1 and R-11 culture filtrate stock solution significantly reduced the number of SR sclerotia by 75.64 and 86.91%, respectively. Additionally, they showed a strong inhibitory effect on the sclerotium germination. The control PDA plate showed 2.67% of non-germinating sclerotium, while the plates containing F-1 or R-11 culture filtrate stock solution showed strong inhibition of sclerotium germination reducing to 96.33% and 88.67%, respectively. With the increase of culture filtrate dilutions, the inhibition effect decreased gradually; however, it remained significantly higher than the control treatment. Additionally, volatile compounds generated by F-1 and R-11 significantly inhibited the growth of SR mycelium by (97.56 ± 1.02)% and (85.07 ± 2.55)%, respectively, showing a robust inhibitory activity against the fungi (Fig. S2).

### Pot experiment

As shown in Table 3, peanut plants inoculated with only stem rot pathogen were seriously ill, with the infection rate reaching 96.67% and the disease index of 73.33. Inoculation with antigenic strains F-1 and R-11 significantly reduced the infection rate and disease index of peanut plants. After 28 d of treatment, the relative control effects of F-1 and R-11 fermentation broth on peanut stem rot were 77.13% and 64.78%, respectively. This was significantly higher than the carbendazim treatment (35.22%, *P* < 0.05).

**Table 3:** Biocontrol performance of antagonistic strains against peanut stem rot

Treatment	Morbidity (%)	Disease index	Control efficacy (%)
−ve control	0 e	0 e	--
+ve control	96.67 ± 4.71^a^	73.33 ± 4.25^a^	--
Fungicide	66.67 ± 4.71^b^	47.50 ± 2.04^b^	35.22 ± 1.40^b^
F-1	30.00 ± 0^d^	16.67 ± 1.18^d^	77.13 ± 2.71^a^
R-11	40.00 ± 0^c^	25.83 ± 1.18^c^	64.78 ± 2.81^a^

Treatments: −ve control = no inoculation; +ve control = pathogen inoculated; Fungicide = carbendazim + pathogen; F-1 = *Bacillus* sp. F-1 + pathogen; R-11 = *Burkholderia* sp. R-11+ pathogen. Different lowercase letters in the same column indicate a significant difference between the treatments (*P* < 0.05)

### Antagonistic bacteria promoted systemic resistance in peanuts

**Fig. 2:**
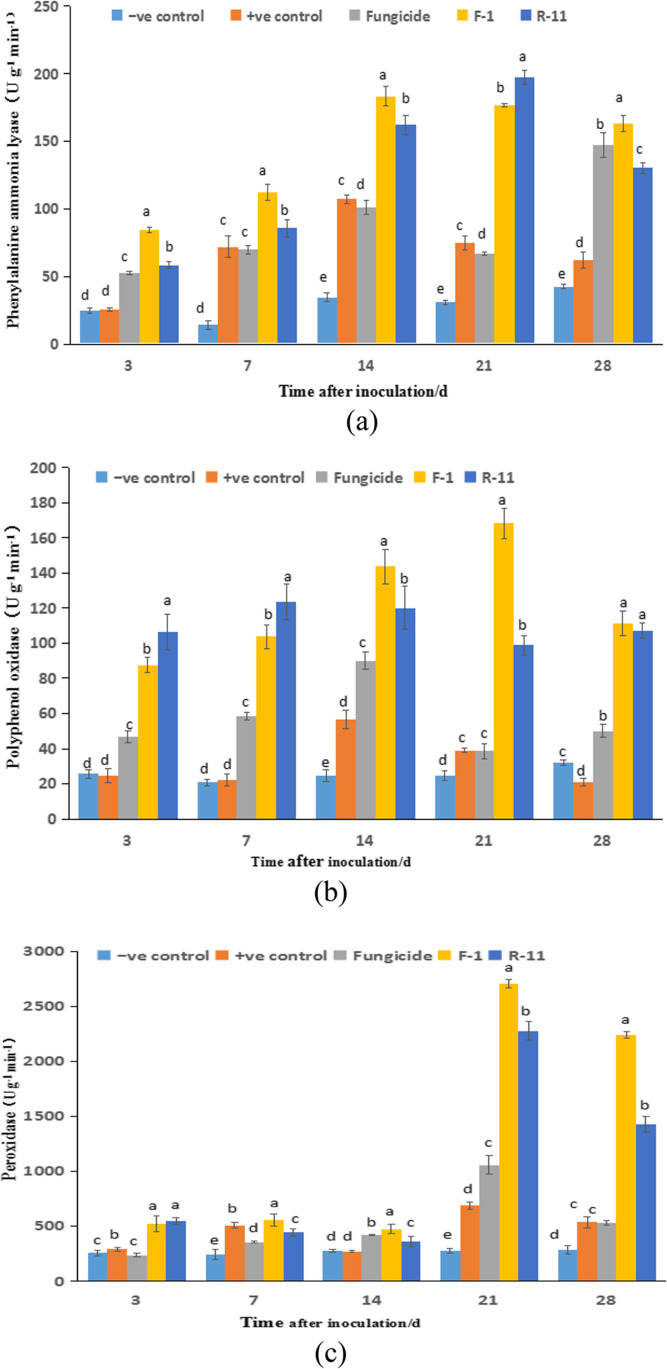
Effects of different treatments and inoculation times on the defense enzyme activities in peanut leaves. (**a**) Phenylalanine ammonia-lyase (PAL), one enzyme activity unit denotes the change of absorbance value (290 nm) of 1 g tissue by 0.01 in one minute. (**b**) Polyphenol oxidase (PPO), one enzyme activity unit denotes the change of absorbance value (410 nm) of 1 g tissue by 0.01 in one minute. (**c**) Peroxidase (POD), one enzyme activity unit denotes the change of absorbance value (470 nm) of 1 g tissue by 0.01 in one minute. Different lowercase letters above the error bars indicate a significant difference between the treatments (*P* < 0.05)

After inoculating the pathogen, we measured PAL, PPO, and POD activity of different treatment groups in peanut leaves at 3, 7, 14, 21, and 28 d. The PAL activity in all treatment groups first increased, then decreased, and was maximum on the 14th and 21st days (Fig. 2a). Additionally, the enzyme activity was significantly higher in the F-1 and R-11 treatment groups than in the carbendazim group (*P* < 0.05), indicating an F-1 and R-11 mediated induced effect on PAL activity.

Figure 2b illustrates the change in PPO activity in different treatment groups at different growth stages. The PPO activity reached its peak in the carbendazim treatment group on the 14th day (90.08 U g^-1^ min^-1^) and then decreased rapidly. The PPO activity in the R-11 treatment group reached the maximum (23.45 U g^-1^ min^-1^) on the 7th day and then decreased slowly. The PPO activity in the F-1 treatment group continued to rise from 3 to 21 d, reaching the maximum of 168.36 U g^-1^ min^-1^. Overall, F-1, R-11, and carbendazim treatments significantly improved the PPO activity in peanut leaves in the order of F-1 > R-11 > carbendazim.

Figure 2c shows the change in POD activity in different treatment groups at different growth stages of peanut leaves. In all treatment groups, POD enzyme activity was relatively stable from 3 to 14 d. After 21 d, the enzyme activity increased rapidly in the F-1, R-11, and carbendazim treatment groups reaching the maximum of 2708.76, 2276.02, and 1059.66 U g^-1^ min^-1^, respectively, which was significantly higher than the 687.42 and 275.89 U g^-1^ min^-1^ of control groups. These results demonstrated that F-1 and R-11 continuously induced POD activity enhancing the stress resistance in peanut plants.

## Discussion

Plant endophytic bacteria, with unique abilities to colonize plant tissues, inhibit pathogens, and promote plant growth, are considered an excellent biocontrol resource. These have also been isolated from different tissues in peanuts to examine their biocontrol potential. For instance, Wang and Liang [31] isolated 37 endophytic bacteria strains from healthy peanut plants and found that *B. amyloliquefaciens* BZ6-1 has a strong antagonistic effect on the pathogen *Ralstonia solanacearum*. *R. solanacearum* infected peanut plants inoculated with 15 mL (10^8^ cfu mL^-1^) of BZ6-1 fermented broth showed a decrease in morbidity to 12.1%. Peanut stem rot is a soil-borne fungal disease caused by SR restricting peanut production. The prevention and treatment of peanut stem rot disease are challenging. Nevertheless, isolation of peanut endophytic bacteria for the prevention/treatment of peanut stem rot has been seldomly reported. Accordingly, in this study, endophytic bacteria strains were isolated from root, stem, leaf, and flower tissues of peanut; six of them showed strong antagonistic potential against SR. Among them, the culture filtrate of F-1 and R-11 showed the best effect with the inhibition rate of 91.66 and 90.56%, respectively. These two were identified as *Bacillus* sp. and *Burkholderia* sp., respectively. *Bacillus* sp. is a dominant soil bacterium with strong stress resistance and broad-spectrum antibacterial activity. *Bacillus* sp. has shown broad application prospects in agriculture controlling plant diseases, promoting crop growth, and improving crop yield [32]. Prevention and treatment of peanut stem rot using *Bacillus* sp. have been widely reported. For instance, Yang [33] reported the isolation of *B. subtilis* Y14 from the peanut rhizosphere that significantly reduced the plant morbidity, improved the rhizosphere environment, increased the number of beneficial bacteria, and promoted the growth and development of peanut plants. *Burkholderia* sp., a widely existing microbe in soil, water, and plant rhizosphere, have been explored for biological control, biodegradation, plant growth promotion, and other functions [34]. It showed biological control effects against various pathogens, including *Penicillium expansum* [35], *Colletotrichum musae* [36], *Sclerotinia laxa* [37], and *Botrytis cinerea* [38]. However, it has not been explored for the prevention and treatment of peanut stem rot. To the best of our knowledge, this is the first study to report the *Burkholderia* sp. antagonistic effect on SR as a biological control agent for peanut stem rot.

Sclerotium is a unique asexual reproductive structure of SR, which has strong adaptability to extreme environments making it challenging to cure SR disease [39]. Errakhi et al. [40] reported the isolation of two *Streptomyces* strains (J-2 and B-11) from beet rhizosphere soil with antagonistic activity against SR. These two strains showed 100% inhibition of sclerotium germination; however, their effect on sclerotium formation in SR is unknown. This study screened antagonistic strains F-1 and R-11 with potent inhibitory effects on the formation and germination of SR sclerotium*.* The inhibition of sclerotium germination effectively reduces its infection rate, while the inhibition of sclerotium formation effectively reduces the number of pathogen bacteria in the field reducing the reinfection rate.

Additionally, volatile compounds generated by F-1 and R-11 produced a strong inhibitory effect on the growth of SR mycelium with inhibition rates of 97.56 and 85.07%, respectively. Compared with culture filtrate, volatile compounds showed better inhibition of mycelium growth. This is consistent with a study showing volatile compounds generated by *Bacillus siamensis* ZHX-10 were superior to culture filtrate against SR [41]. Inhibition or antagonism through the production of volatile compounds by endophyte has been earlier reported, such as in the fungus *Muscodor*. One of the most abundant volatile compounds generated by *Muscodor* is cyclohex-3-en-1-ol and β-bisabolol that act against a broad spectrum of fungi [42, 43]. At present, it is mainly *Trichoderma* that can produce volatile compounds against SR. Rajani et al. [44] demonstrated that volatile compounds play a major role in antagonism of pathogenic fungi by four endophytic fungi belonging to the genus *Trichoderma*. The double-plate assay showed that all the four endophytic *Trichoderma* species significantly inhibited mycelial growth of SR. Head-space analysis of the volatile compound of *T. longibrachiatum* revealed the presence of a large class of compounds including hydrocarbons, ketones, esters, and different classes of terpenes. Sridharan et al. [45] reported *T. longibrachiatum* EF5 inhibits the growth of SR via the production of the volatile compounds. The volatile compounds reduced mycelial growth and inhibited the production of sclerotia by altering the mycelial structure. GC–MS results revealed that EF5 emitted terpenoid compounds, such as caryophyllene, cedrene, and cuprenene. At present, there are few reports on volatile compounds against SR produced by bacteria. Therefore, in the future, we plan to focus on volatile compounds generated by endophytic bacteria F-1 and R-11.

Furthermore, we investigated the prevention and treatment effects of F-1 and R-11 on peanut stem rot in a greenhouse setting. F-1 and R-11 significantly reduced the morbidity and severity of peanut stem rot. Their control effects were significantly higher (77.13% and 64.78%, respectively) than that of the fungicide control group (35.22%, *P* < 0.05). Plant endophytic bacteria protect plants from pathogens through a variety of mechanisms, including the promotion of plant systemic resistance and defense signals [46]. The induced systemic resistance refers to the process of physiological and biochemical changes that activates the plant defense genes [47]. Apart from the change in plant cell wall structure, some other physio-biochemical changes include the upregulation of enzyme activities such as PAL, POD, and PPO [48]. This study found that F-1 and R-11 treatment upregulated the activity of the first key enzyme PAL and terminal enzymes POD and PPO in phenylpropane metabolism in peanut leaves. This could have induced the biosynthesis of resistant substances such as lignin, phytoalexin, and phenols, improving peanut resistance to SR infection. However, the effect of F-1 and R-11 inoculation on other growth indices (e.g., root, plant height, and fresh weight) of peanuts must be further studied.

In conclusion, the present study described two peanut endophytic *Bacillus* sp. (F-1) and *Burkholderia* sp. (R-11) antagonism against SR and evaluated their biocontrol efficacy under greenhouse conditions. We found these bacteria protect the peanut plant from SR infection directly by producing soluble and volatile compounds to inhibit hyphae growth, sclerotia formation, and germination of SR, and meanwhile, indirectly by inducing a peanut plant systemic resistance. In a greenhouse experiment, applying the F-1 and R-11 treatments significantly reduced the morbidity and severity of peanut stem rot (*P* < 0.05), obtaining significantly higher biocontrol efficacy compared with the fungicide treatment. In the future, field experiments should be carried out to study the biocontrol effect of F-1 and R-11 on peanut stem rot and explore their specific application methods.

## Supplementary Materials

ESM 1 (DOCX 828 kb)

ESM 2 (DOCX 13 kb)

ESM 3 (DOCX 462 kb)
