# Supplementary material for: The antagonistic potential of peanut endophytic bacteria against Sclerotium rolfsii causing stem rot
Source: Braz J Microbiol. 2022 Dec 27;54(1):361–70. doi: 10.1007/s42770-022-00896-x (PMC9944171; doi:10.1007/s42770-022-00896-x)
Supplement: Supplementary file 1 — (DOCX 828 kb) [file 42770_2022_896_MOESM1_ESM.docx]

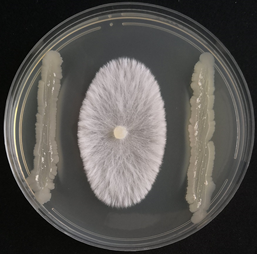

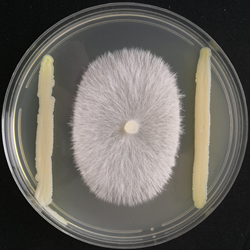


b

c


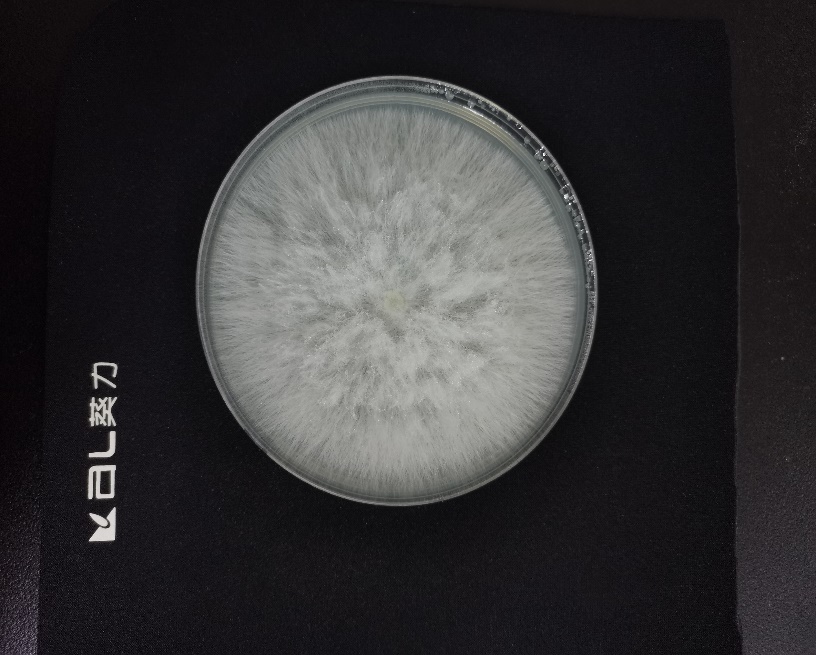


a

**Fig. S1** Antagonistic effects of peanut endophytic bacteria against *Sclerotium rolfsii*. A PDA plate was covered with *S. rolfsii* (a) without inoculating antagonistic bacteria, (b) after inoculation with Strain F-1, and (c) after inoculation with Strain R-11. Both F-1 and R-11 inhibited the growth of *S. rolfsii*.
