# Supplementary material for: The antagonistic potential of peanut endophytic bacteria against Sclerotium rolfsii causing stem rot
Source: Braz J Microbiol. 2022 Dec 27;54(1):361–70. doi: 10.1007/s42770-022-00896-x (PMC9944171; doi:10.1007/s42770-022-00896-x)
Supplement: Supplementary file 2 — (DOCX 13 kb) [file 42770_2022_896_MOESM2_ESM.docx]

**Table S1** Physiological and biochemical characteristics of F-1 and R-11

| Characteristics | F-1 | R-11 |
| --- | --- | --- |
| Gram staining | + | - |
| Anaerobic | - | - |
| Catalase | + | - |
| Lecithinase | + | - |
| Gelatin liquidized test | + | - |
| Starch hydrolysis | + | - |
| Citrate utilization | - | + |
| Indole test | - | - |
| Methyl red test | + | - |
| V-P test | - | + |
| Acid production | | |
| Glucose | + | + |
| Arabic candy | + | + |
| Mannitol | + | + |
| Soluble starch | + | - |
| Sucrose | + | + |
| Maltose | + | + |
| Growth in NaCl | | |
| 2% | + | + |
| 5% | + | + |
| 7% | + | - |
| 10% | - | - |

Note: + Positive; − Negative.
