# Supplementary material for: The antagonistic potential of peanut endophytic bacteria against Sclerotium rolfsii causing stem rot
Source: Braz J Microbiol. 2022 Dec 27;54(1):361–70. doi: 10.1007/s42770-022-00896-x (PMC9944171; doi:10.1007/s42770-022-00896-x)
Supplement: Supplementary file 3 — (DOCX 462 kb) [file 42770_2022_896_MOESM3_ESM.docx]

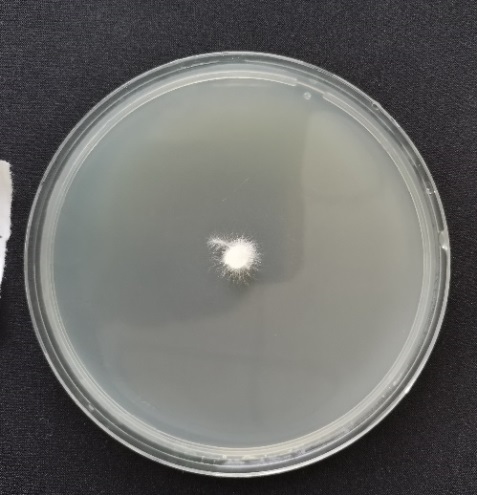

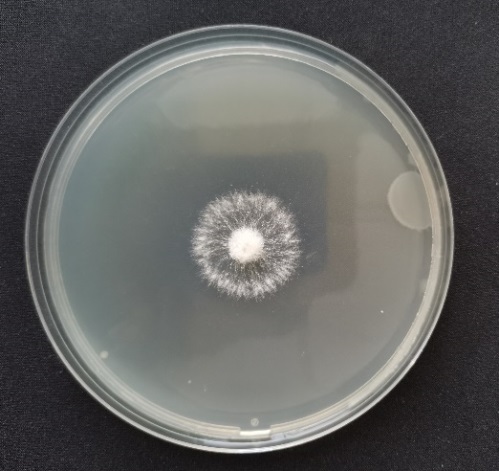


b

c


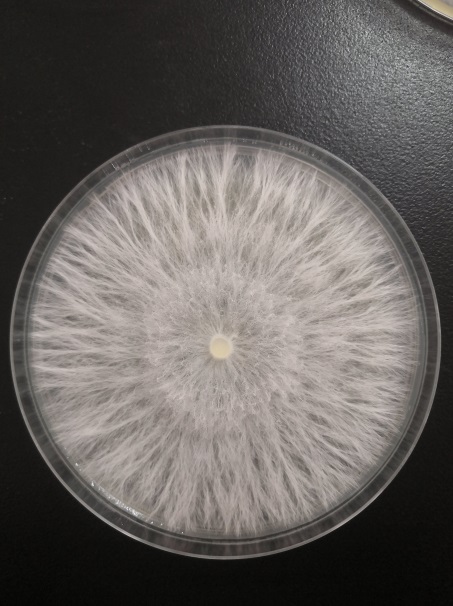


a

**Fig. S2** The inhibitory effect of volatile compounds against *Sclerotium rolfsii*. (a) Without the inoculation of antagonistic strains, *S. rolfsii* overgrew the PDA plate. In the presence of (b) F-1 and (c) R-11 inoculation, the radial growth of *S. rolfsii* was significantly inhibited.
